# Exposure to “Exergames” Increases Older Adults’ Perception of the Usefulness of Technology for Improving Health and Physical Activity: A Pilot Study

**DOI:** 10.2196/games.4275

**Published:** 2015-11-27

**Authors:** Marie-Louise Bird, Brodie Clark, Johanna Millar, Sue Whetton, Stuart Smith

**Affiliations:** ^1^ Healthy Research Centre Faculty of Health University of Tasmania Launceston Australia; ^2^ University of the Sunshine Coast Sippy Downs Australia

**Keywords:** health care reform, postural balance, pleasure, exercise, perception

## Abstract

**Background:**

High rates of sedentary behaviors in older adults can lead to poor health outcomes. However, new technologies, namely exercise-based videogames (“exergames”), may provide ways of stimulating uptake and ongoing participation in physical activities. Older adults’ perceptions of the use of technology to improve health are not known.

**Objective:**

The study aimed to determine use and perceptions of technology before and after using a 5-week exergame.

**Methods:**

Focus groups determined habitual use of technology and the participant’s perceptions of technology to assist with health and physical activity. Surveys were developed to quantitatively measure these perceptions and were administered before and after a 5-week intervention. The intervention was an exergame that focused on postural balance (“Your Shape Fitness Evolved 2012”). Games scores, rates of game participation, and enjoyment were also recorded.

**Results:**

A total of 24 healthy participants aged between 55 and 82 years (mean 70, SD 6 years) indicated that after the intervention there was an increased awareness that technology (in the form of exergames) can assist with maintaining physical activity (*P*<.001). High levels of enjoyment (Physical Activity Enjoyment Scale [PACES-8] score mean 53.0, SE 0.7) and participation rates over the whole study (83%-100%) were recorded.

**Conclusions:**

Older adults’ have low perception of the use of technology for improving health outcomes until after exposure to exergames. Technology, in the form of enjoyable exergames, may be useful for improving participation in physical activity that is relevant for older adults.

## Introduction

Adequate levels of physical activity are a primary factor contributing to the maintenance of physiological and psychological health, yet many older adults are physically inactive [1-3]. Insufficient levels of physical activity have been reported in as much as 62% of the older population (ie, not meeting the recommended guidelines of 30 minutes moderate activity on most days of the week) [4]. As well, they also reportedly spend a major proportion of their day in activities that use very little energy expenditure and would be classified as sedentary. Sedentary behavior levels in Australia are reported to be as high as 40% for adults aged between 65 and 74 years [3,5]. The magnitude of this health issue is set to increase with the changing demographics of our population with the Australian Bureau of Statistics [6] predicting that the portion of the population aged 65 years and older will increase to 29% by 2051.

The health benefits of adequate levels of physical activity and regular exercise include improvements in lower limb strength, balance, and mobility, which may provide a reduction in the risk of accidental falls in older adults. As well, a reduction in incidence in a range of chronic health conditions is seen with changes from sedentary behaviors to more active behaviors. Overcoming barriers and identification of facilitators that will improve uptake of positive health behaviors and encourage long-term participation of such behaviors forms the thrust of current population-based health research.

Advances in technology, both hardware and software, has enabled increased accessibility of technology-based exercise interventions to a vast number of consumers [7]. The design of exercise-based videogames (“exergames”) provides activities that balance enjoyment, ability, and intensity levels to a large market audience [8]. Enjoyment of an activity has been identified as one of the predictors of the effectiveness of an exercise program and, because of this, interactive exercise-based technology, or exergaming, is becoming an increasingly popular strategy for the implementation of physical activity [7-9]. Incorporating the use of interactive games into home-based exercise programs addresses several access barriers around transport and leaving the home, while at the same time providing enjoyable activities may improve ongoing participation in physical activities [7]. The feasibility of trialing exergaming interventions in an older population will rely on acceptance of this type of technology, but evidence about older adults’ attitudes toward using technology to improve physical activity and health more generally is mixed.

The assumption that interest in technology decreases with age is misleading [10] and the small amount of literature available in this area is conflicted. Several studies report negative attitudes and limited use of technology by older people [11,12], whereas others report that older people are enthusiastic about the potential for eHealth and are increasingly adopting these technologies [13-15]. Despite this diversity in findings, there is strong evidence to suggest that older adults are more likely to use applications that they perceive as being user-friendly, engaging, and meeting a current need [13,15]. Miller et al [16], in their review of the literature focusing specifically on home-based exergaming systems used by older adults, suggest that the evidence supporting positive benefits is “relatively weak, with a high risk of bias.” However, older adults have been reported to find exergaming appealing [17] and that it provides improved motivation for activity [18]. As well, perceived enjoyment has been correlated to improved physical well-being during an exergame intervention [19].

From the age of 45 years onward, balance control function declines [20]. Exercise has been shown to improve balance and exercise programs that include a high dose of balance training (without a walking component) reduce fall rates by up to 38% [21]. Although balance training has been recently included in guidelines for exercise for adults older than 65 years [22], many older adults do not participate in balance training as part of their habitual exercise, with only 6% performing balance training and 27% undertaking balance-challenging activities [23]. A recent review of exergames to improve balance in older adults found improvements in at least one facet of postural balance occurred in 10 of 13 of them [24]. Although many of these studies were small, this provides some evidence that balance-related exergames may be useful in assisting older adults meet this component of the exercise guidelines.

The primary aim of this study was to determine the perceptions of the use of technology for health before and after the use of an exergame intervention designed to improve postural balance and to record perceptions of enjoyment after the intervention.

## Methods

The small number of participants involved in this pilot project and the exploratory nature of the research lent itself to a methodology that enabled researchers to explore participant responses in some depth. Heinz et al [25] suggested that opinions of technological developments can be achieved through focus group research, where researchers can relatively easily gain information from older adults. Therefore, a qualitative descriptive study was utilized based on data analysis from 3 focus groups [26].

### Participants

Eligibility criteria of the participants included targeting older adults (>50 years), classified as low risk according to the American College of Sports Medicine guidelines, and currently participating in a previously established Pilates program established at the Exercise Physiology Clinic at the Newnham campus of the University of Tasmania. No participants were excluded.

### Procedure

Potential participants were invited to participate in a focus group held 1 week before initiation of the 5-week exergame intervention. From this focus group, a survey was developed and administered before and after the intervention.

### Focus Group

The focus group was run as an open discussion forum with one experienced researcher directing the group and asking questions, while another trained research assistant took notes and recorded the session for later analysis. Structured open-ended questions were used in the focus groups to elicit information regarding current use and access to technology and the types of technology that this cohort currently engaged with. The whole team was involved in the development of the questions; this has been shown to enhance the validity of the research in the design stage [27]. Current physical activity and perceptions of the impact on physical activity when using technology were explored. The forum was designed to gather qualitative information regarding each person’s current health and physical activity levels, their reasons for exercise, and their technology use and knowledge. This included perceptions and awareness of using technology for health. This information was used to develop a questionnaire by the research team that quantitatively assessed perception of how useful technology can be for a range of health parameters and physical activity: the Technology Engaging Activity (TEA) questionnaire. Pretesting of questions was undertaken to ensure validity of survey questions. Before commencing the intervention, this questionnaire was administered and participants were invited to familiarize themselves with the intervention.

Throughout the study, the intervention was set up as a 2-minute station during the Pilates classes for voluntary and independent access by the participants at any time throughout the hour-long timeframe supervised by 2 research assistants. The technology was available for 5 weeks, which included a total of 10 Pilates sessions. After the conclusion of the intervention, participants were invited to complete the same questionnaire and the 8-item Physical Activity Enjoyment Scale (PACES-8) questionnaire. The exergame was introduced as an individual 2-minute station in a circuit class (see Multimedia Appendix 1). This allowed for maximum engagement of the exergame, but did not require any extra effort from the participants. Research assistants implemented the program, which was the game “stack ’em up” contained within the “Your Shape: Fitness Evolved 2012” (Ubisoft) exergame package for the Microsoft Xbox 360 Kinect game console. The level of difficulty throughout the intervention was set at easy and was not adjusted throughout the study. Game scores were collected for each participant after the game was completed. Comments while performing the game were collected by the researchers.

**Figure 1:**
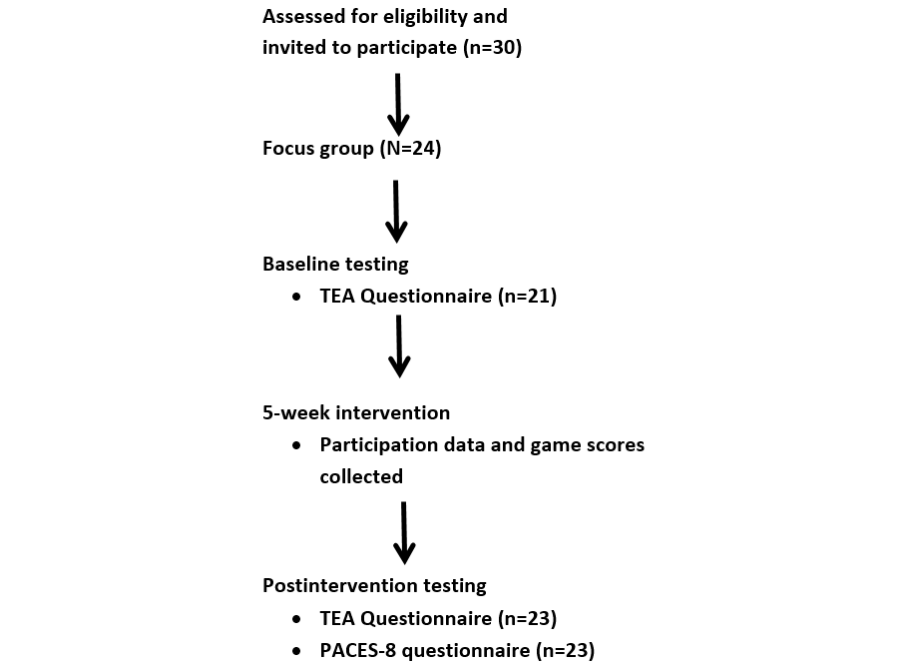
Study flow diagram.

Ethics approval from the Social Science Human Research Ethics Committee (H0013878) was gained before commencement of the study. Participant flow through the study and data collection time points are outlined in Figure 1.

### Measures

#### Perceptions of Technology Enhancing Physical Activity

Participant’s perceptions of technology usage were gauged through the TEA questionnaire. This questionnaire was composed of 5 questions and responses were measured on a Likert scale of 1 to 5, in which a score of 5 represented a strong agreement and a score of 1 indicated a strong disagreement. This was developed by the research team and established from participants’ responses in the focus group. Two research assistants administered the survey. Specifically, the questionnaire asked participants to rate their level of agreement/disagreement with the following statements:

1. I think technology can keep me active.
2. I think technology is useful in my life.
3. I think technology is enjoyable.
4. I think technology helps me be more active.
5. I think technology can improve my postural balance.

#### Exergame Enjoyment

The PACES-8 [28] is a validated and reliable modified version of the original 18-item PACES questionnaire, which describes enjoyment of physical activity within the older adult population. For each of the 8 questions, a score of 7 represents maximal enjoyment and a score of 1 represents minimal enjoyment for each subsection, resulting in a total possible score of 56.

### Data Analysis

The data analysis drew on semantic thematic analysis to identify explicit surface meanings within the data. Thematic analysis is appropriate for questions seeking to explore people’s views or perceptions [29,30]. Because this evaluation sought user opinions and perceptions, the methods of thematic analysis were considered appropriate.

Thematic analysis of focus group data was undertaken from both recordings and notes from the sessions using a phenomenological approach. Recordings were analyzed in a group session. Each researcher listened to the material and individually noted common responses. These were then discussed as a group until consensus about common patterns was reached and these were used as a basis for manual coding of data. In addition to identifying common patterns, the range of views for each pattern was identified with examples across the spectrum recorded as anonymous quotations.

Microsoft Excel was used to analyze the quantitative data from the surveys, which was reported as mean and standard error of means. Pre- and postintervention data were analyzed using paired *t* tests.

## Results

### Participants

A total of 24 participants (5 male, 19 female) aged between 55 and 82 years (mean 70, SD 6 years) were recruited to participate in the focus groups and technology engaging intervention for older adults study. Although the number of attendees participating in the exergames session in the circuit class varied over the duration of the intervention, participation rates increased from week 1 (20/24, 83%) to all participants in week 4 (24/24, 100%) with a slight drop seen in week 5 back to 21 participants (88%).

### Focus Group

The focus group identified that this active group of older adults primarily used technology for pragmatic purposes and the majority indicated little exposure to using technology for enjoyment (23/24) or games (18/24). In fact, the game-based technology that they currently engaged in encouraged sedentary behaviors.

The focus group established that, before exposure to the intervention, the majority (23/24) of participant’s engagement with technology was for mainly pragmatic reasons, such as communication (eg, mobile phones, email, and use of Skype to communicate with family members) and simple information gathering (eg, timetables and location of services). Although this majority indicated that technology was “not used for enjoyment” and “only do what I need to do,” a few people (2/24) identified enjoying interacting with new technology and provided positive responses such as “I’m a gadget baby” and “very useful when needed.”

When asked about technology and games, participants only identified participating in technology-based games, such as Solitaire and FreeCell. Generally, participants seemed unaware that it was possible to use gamed-based technology for improving health outcomes, indicating that the computer-based games they currently participated in reduced activity and were not positively related to health. Two of 24 participants indicated that they had at one time (but did not regularly) played with a Nintendo Wii console with their grandchildren.

Participants indicated strong engagement in a variety of exercise activities over many years. It was established that participants engaged in both structured and unstructured exercises daily and mostly of moderate intensity. Many in the group described their preferential involvement toward exercise in a social environment (eg, dancing, swimming, and bushwalking groups), whereas other participants focused on more individual activities (eg, gardening, walking, and riding) with each participant indicating that the autonomy of exercise selection enhanced participation. Participants described that they engaged in multiple types of physical activity throughout the week, including both social and individual activities regularly. Participants expressed that exercise in their life was related to being “habitual” and to maintain or improve their health.

### Perceptions of Technology Enhancing Physical Activity

Pre- and postsurvey data indicated that participants significantly increased their positive perceptions of the use of technology to keep active and improve postural balance (*P*<.05) (Table 1).

**Table 1:** Survey responses pre- and postintervention (N=24).^a^

Statement	Preintervention, mean (SE)	Postintervention, mean (SE)	*P*
I think technology can keep me active	2.95 (0.21)	4.00 (0.23)	<.001
I think technology is useful in my life	3.90 (0.17)	3.94 (0.19)	.44
I think technology is enjoyable	3.48 (0.21)	4.00 (0.21)	.04
I think technology can help me be more active	3.38 (0.23)	3.78 (0.22)	.05
I think technology can improve my postural balance	3.76 (0.17)	4.22 (0.19)	.03

^a^ Based on a 5-point Likert scale (5=strongly agree, 1=strongly disagree).

### Exergame Enjoyment

The postintervention PACES-8 enjoyment questionnaire focused solely on the chosen exergame used throughout the intervention. Participant mean results identified that all the questions received a score of 6 or higher. From the PACES-8 questionnaire, an overall score of 56 signifies the maximal score that can be achieved per individual. The mean pooled response from participants was 53.0 (SE 0.7).

### Game Scores

Game scores increased from week 1 (mean 892, SE 65) to week 5 (mean 1579, SE 112). Researchers observed that participants endeavored to increase their scores over the time of the study.

## Discussion

The primary aim of this study was to determine the perceptions of older adults to technology for health before and after participating in an exergame intervention. This group initially indicated commitment to nontechnology-based physical activity; however, a significant change in attitude was seen after the intervention with improvements in understanding about the health and activity benefits of using technology in the form of exergames. High levels of enjoyment and perceived personal benefit were also identified. This study adds to the evidence supporting the use of exergames as enjoyable and engaging methods for older adults to improve participation in physical activity.

### Pragmatism to Participation

The responses from the focus group identified strong emerging themes associated with attitudes toward the use of technology for pragmatic purposes and the participants’ attitudes about life-long commitment toward physical activity. Although there are positive health outcomes associated with digital video gaming for older adults, our participants were not aware of this before the intervention [31]. When initially questioned regarding their perceptions about technology and games, all participants immediately responded with the idea that these involved limiting physical activity. The impact of sedentary behaviors associated with screen-based technology use is a concern across the life span [32].

### Technology and Activity

Before the intervention, there was a lack of familiarity in these older adults with the concept of utilizing technology as a form of exercise. There were positive changes to responses on both items on the questionnaire relating to physical activity after the intervention. After the intervention, participants indicated that they thought that technology was able to assist in maintaining physical activity levels. There was an increase in the perception that technology was useful to improve physical activity, but this difference did not meet statistical significance (*P*=.05). Specifically, with respect to postural balance, the usefulness of this form of exercise was perceived to increase. The use of technology to enhance physical activity in older adults is receiving attention in current literature as a motivator for improving physical activity, especially to meet the needs of that part of the population.

There was a strong response that indicated maintaining health was a key reason to exercise. One participant stated they felt that “exercise is a part of life,” with the other 7 people in that group affirming that concept. Another participant in a different group described exercise in their life as “habitual”. Although it was identified that the focus group participants used some form of technology on a daily basis, there was only limited exposure to any form of exergame activity. The literature suggests that older adults are more willing to use technologies that they perceive as meeting a current need in a more convenient way than other options [12,14,33-36]. Before the intervention, participants did not view the exergame as meeting their need for regular exercise. However, this perception changed after their participation in the program.

### Technology, Enjoyment, and Engagement

The high levels of enjoyment recorded by the participants augur well for the future of this form of technology to improve physical outcomes using this modality.

Enjoyment has been identified as an important implementation factor in physical activity programs [37]. As well, the literature suggests that older adults who find enjoyment in physical activities tend to perform them for longer periods of time [19]. Overall enjoyment and levels of satisfaction have been shown to be better predictors of physical activity participation and adherence than any other factors [38,39].

Indication of engagement with this form of exercise is supported by high voluntary participation rates throughout our study. Researchers noted that participants strategically challenged themselves to gain a higher overall score and continued to engage in the exergame over the period of the intervention. The researchers also identified that scores needed to be monitored and recorded for each participant to further challenge the participants and retain and reinforce enjoyment levels.

The social context of this study design (ie, being part of a group and having the ability to compare scores with others) contributed to the engagement of this group in the intervention. Future research needs to identify the types of people who would engage with this technology in their own home, without face-to-face social contact, if we want to use exergames as part of a widespread intervention to overcome many barriers to physical activity that older adults have in leaving their home. Technology that links virtual groups may overcome the potential barrier of social isolation and may be of benefit for both improving physical activity within a virtual social network for those people unable to mobilize easily outside the home. In the future, home-based preventive health care using technology may be leveraged off current research exploring in-home rehabilitation using motion capture software and technology [40].

A limitation of this study was the selection and use of a convenience sample of participants who were currently physical active (ie, attending Pilates classes regularly). This limits the generalizability of these results and precludes application to sectors of the community that are more sedentary and perhaps a better target for interventions such as these. Future research should endeavor to use higher best practice dosage to improve postural balance. Because of the short intervention period, it is important to note that adherence and enjoyment levels may have changed after the 5-week period.

### Conclusion

Exposure to and participation in a balance-focused exergame resulted in older adults dramatically increasing their perception of the usefulness of technology for improving several health outcomes, including physical activity levels and postural balance. High rates of enjoyment and adherence to this program were reported. Technology, in the form of enjoyable exergames, may be useful for improving participation in physical activity that is relevant for the needs of older adults.

## Appendix

Multimedia Appendix 1 (A) Screenshot of intervention for training postural balance. (B) Using the game for training postural balance.

## Abbreviations

PACES: Physical Activity Enjoyment Scale
TEA: Technology Engaging Activity
