# Supplementary material for: Exposure to “Exergames” Increases Older Adults’ Perception of the Usefulness of Technology for Improving Health and Physical Activity: A Pilot Study
Source: JMIR Serious Games. 2015 Nov 27;3(2):e8. doi: 10.2196/games.4275 (PMC4704928; doi:10.2196/games.4275)

Multimedia Appendix 1.

(A) Screenshot of intervention for training postural balance.

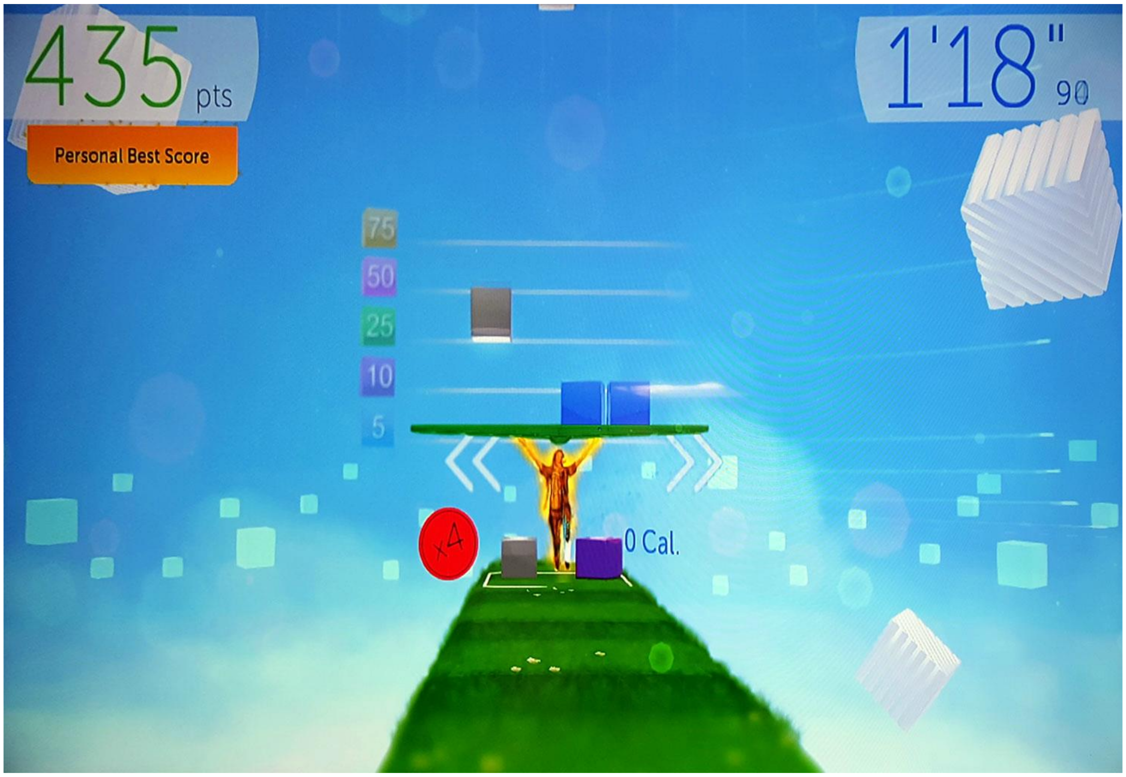

(B) Using the game for training postural balance.

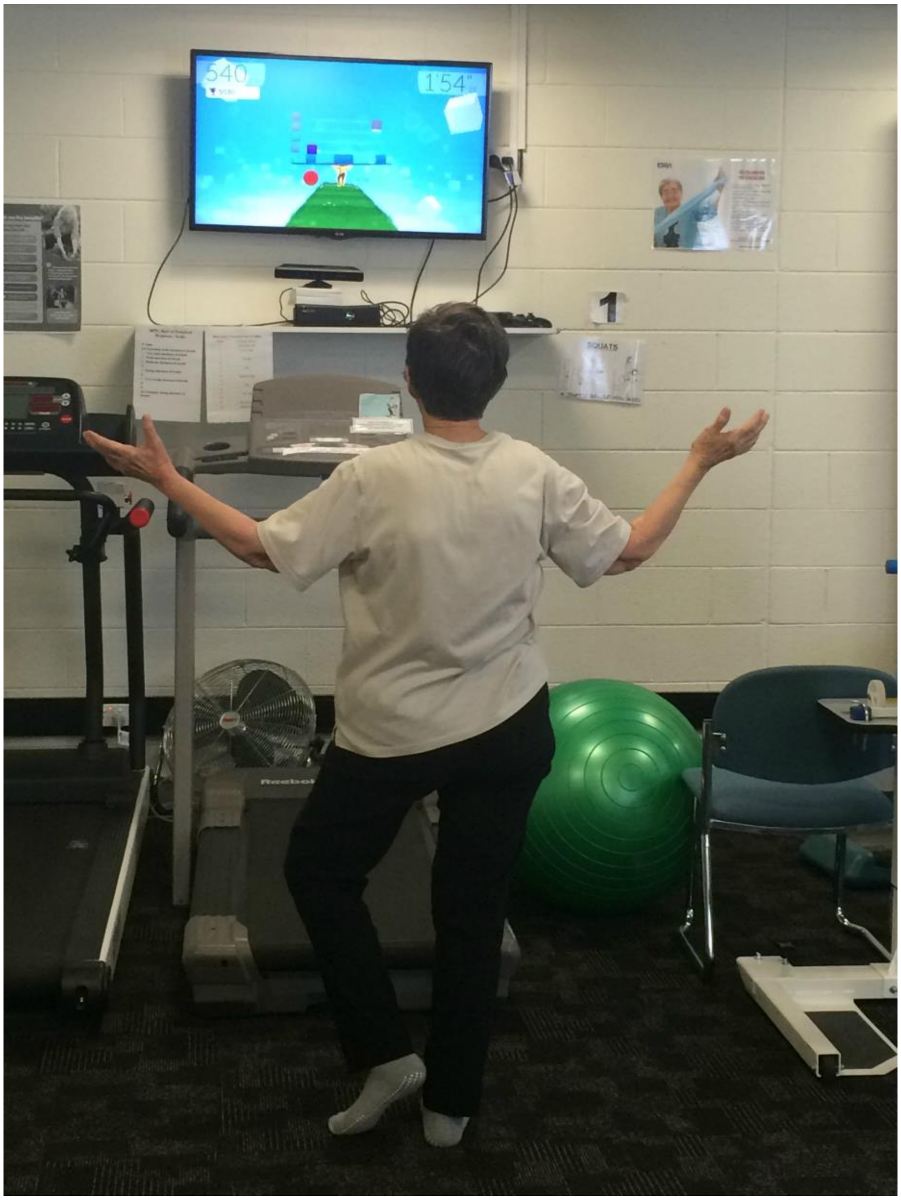

Supplement: Multimedia Appendix 1 [file games_v3i2e8_app1.pdf]
